# Contrast-enhanced ultrasound evaluation of primary renal squamous cell carcinoma: a case report

**DOI:** 10.3389/fonc.2023.1280298

**Published:** 2023-11-24

**Authors:** Yuhao Jia, Qunxia Zhang

**Affiliations:** Department of Ultrasound, The Second Affiliated Hospital, Chongqing Medical University, Chongqing, China

**Keywords:** renal, contrast-enhanced ultrasound (CEUS), squamous cell carcinoma, imaging, diagnosis

## Abstract

Renal squamous cell carcinoma (RSCC) is very rare, and there are few reports about it so far. Here we report a unique case of renal squamous cell carcinoma examined by contrast-enhanced ultrasound(CEUS), which has never been reported before. In addition, the results of CEUS showed some unique features, different from other imaging examinations. The purpose of this case report is to clarify the CEUS findings of this case and analyze its potential value in early diagnosis of RSCC.

## Introduction

Renal squamous cell carcinoma (RSCC) is a very rare malignant urinary tumor. It is highly aggressive with poor prognosis. According to statistics, RSCC accounts for less than 1% of all renal tumors and only 0.5-8% of all urinary tumors (1, 2). Patients with RSCC often present at an advanced stage and are difficult to diagnose at an early stage due to its rare incidence and lack of typical imaging findings. In this manuscript, we attempt to analyze the potential value of contrast-enhanced ultrasound(CEUS) in the early diagnosis of RSCC by describing the contrast-enhanced ultrasound features of RSCC patients and combining relevant literature. To our knowledge, this is the first report on the CEUS findings of RSCC.

## Case report

A 42-year-old male patient complained of severe right flank pain for more than 20 days without obvious cause, characterized by colic without radiating pain. There was no history of weight loss, fever, hematuria, nausea, vomiting or other discomforts during this period. The pain was not significantly relieved after taking household painkillers and anti-inflammatory drugs (the names of these drugs are unknown). The patient was admitted to a local hospital and diagnosed with right nephrolithiasis. A right ureteric stent implantation was performed before discharge. The aforementioned pain gradually worsened subsequent to his discharge from the hospital. Four days ago, the patient went to a higher-level hospital for treatment and underwent enhanced CT scan of the upper abdomen at the hospital. The CT results showed that “after the right ureteral stent placement surgery, there was a stone in the right kidney. The density of the upper and middle portions of the right kidney was abnormal, and infection with abscess formation was considered. The retroperitoneal lymph nodes enlarged, and the tumor remained to be ruled out”. So, the patient came to our hospital for further treatment.

**Figure 1:**
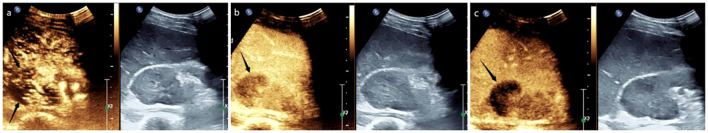
This is the CEUS manifestation of the tumor. **(A–C)** were different enhancement periods from early to late. The overall manifestation is heterogeneous hypoenhancement, Contrast wash in was later than the surrounding parenchyma, while wash out earlier than the surrounding parenchyma, multiple nonenhancing areas could be observed in the lesion.

Physical examination revealed obvious tenderness in the right flank, and the pain was elicited with percussion. Urinalysis showed a white blood cell count of 98/µl. Gray-scale ultrasonography showed that the renal capsule of the upper pole protruded slightly from the surface, and the local parenchyma demonstrated heterogenous echogenicity. A right renal calculus measuring 0.8cm was detected in the upper pole. Color Doppler flow imaging (CDFI) showed poor blood flow signals in the upper pole of the right kidney. The results of renal arteriovenous ultrasound showed no significant abnormalities in the structure, blood flow filling, and blood flow spectrum of both kidneys. Contrast-enhanced ultrasound(CEUS) was then performed to evaluate the microvasculature of the kidney. 1.2 mL of the contrast agent (SonoVue, Bracco SpA, Milan, Italy) suspension was injected through his cubital vein followed by a 5 mL saline flush. The results of CEUS showed a suspected lesion in the upper pole with heterogeneous hypoenhancement, the size was 68 × 36mm. Contrast wash in was later than the surrounding parenchyma, while wash out earlier than the surrounding parenchyma, multiple nonenhancing areas were observed in the lesion (**Figure 1**).

**Figure 2:**
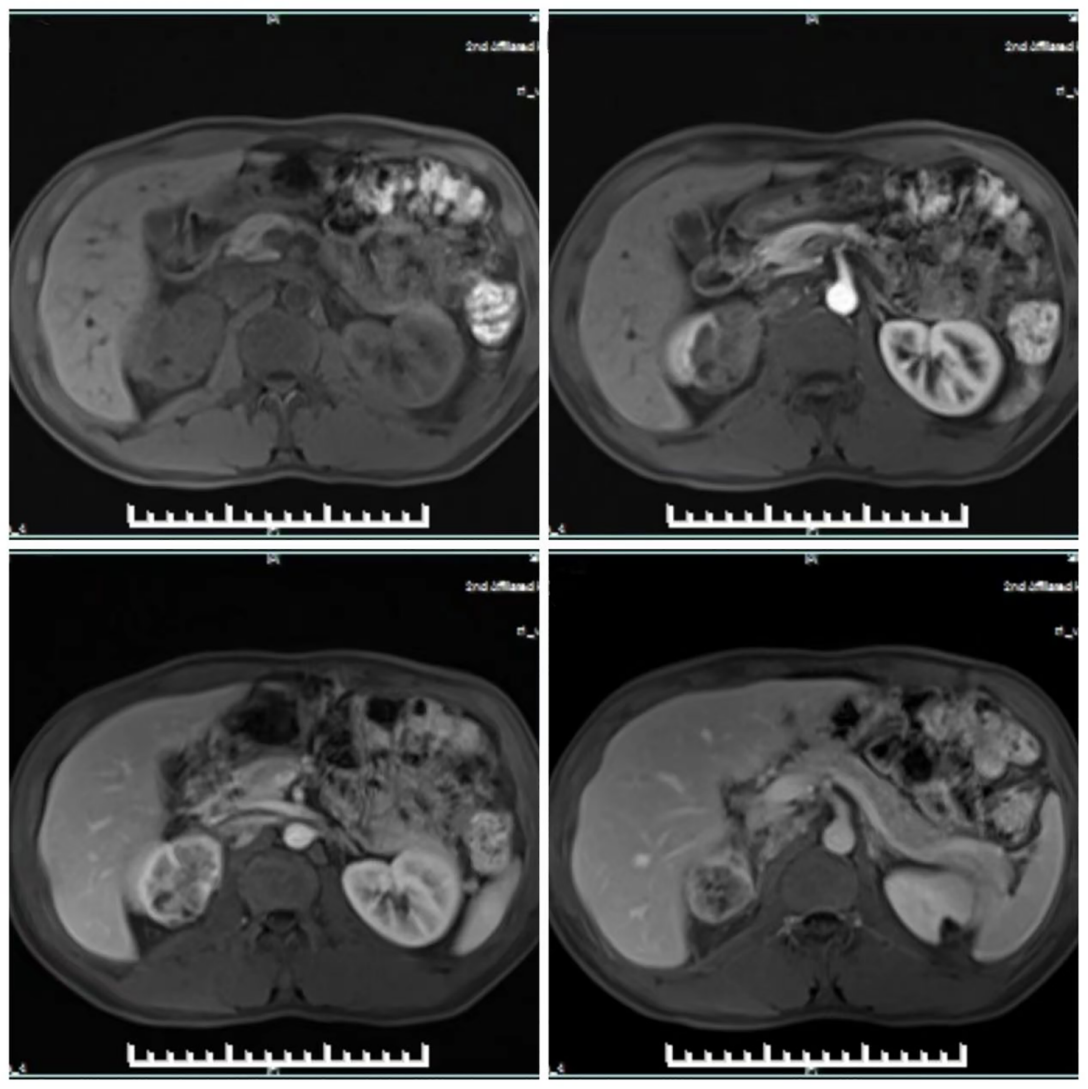
MRI showed heterogeneous enhancement in all phases of enhanced scanning, and multiple small cystic areas without enhancement were found in the tumor.

On abdominal contrast-enhanced CT, an irregular low-density mass with a size of 80 × 50 × 35mm was detected in the upper pole of the right kidney. The upper edge of the lesion protruded slightly from the surface of the kidney. Heterogeneous enhancement was observed in all stages of enhanced scanning, and multiple, small, cystic, non-enhancing areas were seen in the lesion. The lesion involved the adjacent renal calyces and the perinephric fat. Multiple retroperitoneal lymph nodes were enlarged. In addition, similar MRI findings were described (**Figure 2**). Afterwards, the patient also underwent ECT (Emission Computed Tomography) examination, which showed a slight decrease in blood supply to the right kidney, moderate damage to filtration function, and delayed excretion. However, the right upper urinary tract was not obstructed.

The patient underwent laparoscopic right nephrectomy under general anesthesia. During the operation, the upper pole of the right kidney was adherent to the surrounding tissues, the renal hilum adhesion was obvious, and the local enlarged lymph nodes were hard and partially coalescent. Postoperative pathological results showed moderately differentiated squamous cell carcinoma of the right kidney, with involvement of the perinephric fat. Clinical staging: moderately differentiated squamous cell carcinoma of the right kidney (mid-term, T3N1M0).

## Discussion

RSCC is a rare malignant tumor of urinary system with strong invasiveness. It belongs to squamous metaplasia in histology. Its histological features include keratin pearl formation, intercellular bridges and keratinized fragments. However, most RSCCs are moderately or poorly differentiated, so the characteristics of squamous cell carcinoma, such as keratin pearl and intercellular bridge, may not be easily found. Renal squamous cell carcinoma is usually more invasive than most transitional cell carcinomas at diagnosis (3). The occurrence of this disease is usually related to the long-standing renal calculi, especially staghorn calculi. These calculi will lead to chronic irritation and infection of the kidney, and subsequent squamous metaplasia of the renal parenchyma. In this case, the ultrasound results showed that the patient had a stone with a diameter of approximately 0.8cm at the upper pole of the right renal sinus, and the final surgical results confirmed that the tumor in the patient’s kidney was also located at the upper pole of the right renal parenchyma. Therefore, it can be reasonably speculated that the stone is one of the potential causes of differentiated renal squamous cell carcinoma in this case. In addition, hydronephrosis, schistosomiasis, use of chemicals and hormone imbalance are also risk factors of the disease (3, 4). At present, the disease has no characteristic manifestations on routine imaging. Most patients are detected at an advanced stage and have a poor prognosis. Therefore, all signs that may aid early diagnosis are meaningful.

It has been reported that the most helpful features in CT of RSCC are an enhancing extra-luminal and exophytic mass (5). In this case, the patient’s CT showed an irregular mass of low-density in the upper pole of the right kidney and a slightly protruded surface of the kidney, which suggested a space-occupying lesion of the right kidney. Enhanced CT showed heterogeneous, mild to moderate enhancement and multiple small cystic areas without enhancement. It can be inferred that the internal microvessels of the lesion were not rich and accompanied by some tissue necrosis. This enhancement feature has a certain degree of differentiation from clear cell carcinoma, the most common malignant tumor of the kidney, because the microvessels of clear cell carcinoma of the kidney are rich, and the CT enhancement pattern is mostly uniform hyperenhancement (6–8). Compared with other malignant tumors of the kidney, such as chromophobe cell carcinoma and papillary cell carcinoma, these enhancement features could not be used for differential diagnosis, because the enhancement features of chromophobe cell carcinoma and papillary cell carcinoma are similar (6, 8, 9). In conclusion, the diagnostic value of CT alone for renal squamous cell carcinoma is limited because there is no characteristic appearance on CT.

In this case, grayscale ultrasound revealed uneven local parenchymal echo in the upper pole of the right kidney. Studies have shown that this sign is an effective indicator for judging malignant renal masses (10). In addition, CEUS is a promising method for evaluation of microvasculature of lesions. CEUS results showed that the enhancement of the upper pole of the right kidney was heterogeneous, with slow in and fast out hypoenhancement pattern, the results were similar to those of CT scan, and could show more clearly the lesion that was not apparent on conventional- ultrasound. It is worth mentioning that there are differences in the enhancement characteristics between renal malignant tumors and benign tumors. If certain features appear, such as false capsule, uneven enhancement, and “fast in and out” enhancement, the tumor is more likely to be malignant (10). Moreover, previous studies have shown that the enhancement characteristics of various common renal malignancies are also different. For example, the most common renal clear cell carcinoma shows hyperenhancement with fast in and slow out flow pattern, which is similar to the aforementioned CT scan. This manifestation is related to its pathological features. Renal clear cell carcinoma is rich in microvessels, which are tortuous and dense, making it appear rich in blood supply on angiography. Other common types, such as chromophobe cell carcinoma and papillary cell carcinoma, although the contrast results are mostly hypoenhancement, it is mostly homogeneous. However, with the increase of tumor volume (generally > 4cm in diameter), their enhancement is more heterogeneous, which is related to the rapid growth of tumor and internal necrosis (8, 9, 11, 12). The above features belong to the qualitative parameters of contrast-enhanced ultrasound. In addition, contrast-enhanced ultrasound can also analyze the lesion situation through some quantitative parameters, such as were peak intensity (PI), area under the curve (AUC), time to peak (sec) (TP), etc. based on quantitative analysis software. Tufano et al.’s study showed that PI and AUC can provide good assistance in distinguishing benign and malignant renal masses, with accuracy rates of 93% and 95%, respectively (10). This conclusion has also been confirmed in other relevant studies (13). Moreover, Xue et al. validated the reliability of TP in the differentiation of malignant tumors in their report (14). Compared with the CEUS qualitative analysis description in this case, the quantitative analysis will undoubtedly be more objective and accurate. Based on the lack of characteristic imaging findings to assist the diagnosis of RSCC, CEUS quantitative analysis may be a good supplement.

Combining CEUS with CT enhancement results, it is not difficult to find that RSCC in this case is characterized by hypoenhancement due to insufficient microvessels and heterogeneous enhancement due to internal necrosis (large volume and high malignancy). This manifestation is different from other renal malignancies, but it does not have strong specificity.

At present, there is no effective diagnostic method for RSCC except pathological biopsy. It is worth mentioning that research on cell differentiation and microenvironment seems to be of great help for the diagnosis and treatment of kidney tumors, such as cancer stem cells with cloning and differentiation capabilities, as well as tumor microenvironments containing cancer cells and other non-cancer cells, as well as secretion factors (15, 16). Research has shown that cancer stem cells and tumor microenvironment are closely related to the production and development of tumors (15, 16). By intervening with them, effective treatment of tumors can be achieved (16, 17). In addition, cancer stem cells are believed to be the cause of tumor recurrence and metastasis (16). In contrast, contrast-enhanced ultrasound only reflects the blood flow inside the tumor, and its supplementation of information on tumor cell differentiation is limited.

## Conclusion

In summary, compared to existing imaging examinations, contrast-enhanced ultrasound is a valuable supplement for the diagnosis of renal squamous cell carcinoma. The combination of disease risk factors and contrast-enhanced ultrasound imaging features will be more helpful for clinical doctors to diagnose or differentiate the disease. However, due to the rarity of this disease, more cases need to be summarized and analyzed in more detail, such as quantitative analysis of contrast-enhanced ultrasound, in order to obtain more detailed information about the internal blood supply of RSCC.

## Data availability statement

The raw data supporting the conclusions of this article will be made available by the authors, without undue reservation.

## Ethics statement

Written informed consent was obtained from the individual(s) for the publication of any potentially identifiable images or data included in this article.

## Author contributions

YJ: Writing – original draft. QZ: Writing – original draft, Writing – review & editing.
